# Antibacterial Effect of Thiosulfinates on Multiresistant Strains of Bacteria Isolated from Patients with Cystic Fibrosis

**Published:** 2018

**Authors:** V. V. Kulikova, M. Yu. Chernukha, E. A. Morozova, S. V. Revtovich, A. N. Rodionov, V. S. Koval, L. R. Avetisyan, D. G. Kuliastova, I. A. Shaginyan, T. V. Demidkina

**Affiliations:** Engelhardt Institute of Molecular Biology of the Russian Academy of Sciences, Vavilova Str., 32, Moscow, 119991, Russia; N.F.Gamaleya National Research Centre of Epidemiology and Microbiology, Ministry of Health of Russian Federation, Gamaleya, Str., 18, Moscow, 123098, Russia

**Keywords:** allicin, antibacterial activity, cystic fibrosis, methionine γ-lyase, thiosulfinates

## Abstract

The multiresistance of *A. ruhlandii *155B, *B. cenocepacia *122, and *P. aeruginosa *48B strains isolated from patients with cystic fibrosis was established. The antibacterial effect of allicin, dimethyl thiosulfinate, and dipropyl thiosulfinate on multidrug-resistant strains was shown. Thiosulfinates can have both bacteriostatic and bactericidal effects depending on the microorganism and the concentration. The studied thiosulfinates may be candidates for the development of alternative antibiotic drugs to treat infections caused by multidrug-resistant pathogens.

## INTRODUCTION

The emergence of novel approaches to the development of effective antibacterial drugs is of utmost relevance because of the wide spread of antibiotic-resistant strains of bacteria. Multidrug-resistant microorganisms cause nosocomial infections, which can be the origin of complications in weakened patients. The chronic pulmonary infection caused by the association of such pathogens as *Pseudomonas aeruginosa*, *Staphylococcus aureus*, *Burkholderia cepacia complex*, etc. in patients with cystic fibrosis [1] is a serious problem associated with the formation of multi-resistant strains of microorganisms as a result of prolonged antibiotic therapy, which renders further antibiotic therapy ineffective.

Thiosulfinates are found in plants of the genus *Allium *and have an antimicrobial effect [2]. The antibacterial effect of allicin, the main thiosulfinate contained in garlic, is due to the combination of a reduced cellular glutathione level and inactivation of key metabolic enzymes as a result of the modification of their thiol groups [3, 4]. Since allicin, which oxidizes the thiol groups of enzymes and proteins, has many targets within the cell, it, alongside with other thiosulfinates, is unlikely to cause resistance [5].

Alliinase [EC 4.4.1.4] of the plants of the genus *Allium *catalyzes the decomposition of sulfoxides of the S-substituted analogues of *L*-cysteine to give rise to thiosulfinates. We have shown that thiosulfinates can be obtained using methionine γ-lyase (MGL, [EC 4.4.1.11]) (*Scheme*). Thiosulfinates formed by the cleavage of S-allyl-*L*-cysteine, S-methyl-*L*-cysteine, and S-ethyl-*L*-cysteine sulfoxides catalyzed by both wild-type MGL and its more efficient mutant form, C115H, inhibit the growth of Gram-positive and Gram-negative bacteria [6], including *P. aeruginosa *isolated from murine intestine [7].

The aim of the current work was to study the antibacterial effect of thiosulfinates obtained by β-elimination of three S-substituted *L*-cysteine sulfoxides (*Scheme*) catalyzed by C115H MGL on multidrug-resistant strains of the Gram-negative bacteria *Achromobacter ruhlandii *155B, *B. cenocepacia *122, and *P. aeruginosa *48B isolated from patients with cystic fibrosis.

## EXPERIMENTAL

Isolation of the enzyme, determination of its activity, synthesis of S-substituted *L-*cysteine sulfoxides, and production of thiosulfinates were carried out as previously described [6]. The concentrations of thiosulfinates were determined according to [8].

The antibacterial activity of thiosulfinates was determined by the two-fold serial dilution and agar diffusion method.

**Scheme 1:**
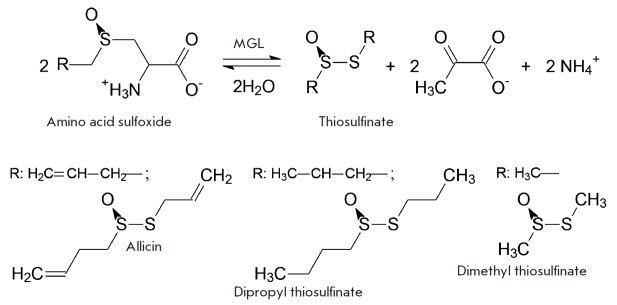
β-elimination reaction of S-substituted *L*-cysteine sulfoxides

When determining the antibacterial activity of thiosulfinates by the method of two-fold serial dilutions [9], we used the Mueller-Hinton broth. Strains were cultivated at 105 CFU/ml and supplemented with agents at concentrations ranging from 1 to 0.0039 mg/ml, followed by inoculation into a dense growth medium (medium no. 1 for *P. aeruginosa *48B and blood agar for *A. ruhlandii *155B and *B. cenocepacia *122).

The antibacterial activity of the agents on the solid growth medium was determined at a concentration varied from 2 to 0.05 mg/ml by inoculating diluted strains (104 to 107 CFU/ml) into Mueller-Hinton agar using the disc diffusion method and by directly applying the test samples at a volume of 10 μl.

Strain resistance to standard antibiotics prescribed to treat cystic fibrosis was determined by the serial dilution method according to the clinical recommendations on the threshold MIC values for each antibiotic [10].

The antibacterial efficacy of the thiosulphinates and antibiotics was compared using the disc diffusion method; strains were inoculated from the diluted cultures (106 CFU/ml) into Mueller-Hinton agar.

The duration of incubation on the solid growth medium was 24–48 h for all the experiments.

Strains *A. ruhlandii *155B, *B. cenocepacia *122, and *P. aeruginosa *48B isolated from patients with cystic fibrosis and stored in the culture collection of the Laboratory of Molecular Epidemiology of Nosocomial Infections at the N.F.Gamaleya National Research Centre of Epidemiology and Microbiology (Ministry of Health of the Russian Federation) were used in this study.

## RESULTS AND DISCUSSION

The antibacterial activities of allicin, dimethyl, and dipropyl thiosulfinate against the *A. ruhlandii *155B, *B. cenocepacia *122, and *P. aeruginosa *48B strains isolated from patients with cystic fibrosis were determined (*Table 1*). The differences in the nature and degree of the antimicrobial effect of the thiosulfinates were revealed.

Allicin and dimethyl thiosulfinate turned out to exhibit the strongest effect against *B. cenocepacia *122 and *P. aeruginosa *48B, while dipropyl thiosulfinate was less active.

**Table 1:** The MIC and MBC values for thiosulfinates

Bacterial strain	Thiosulfinate	MIC	MBC
		mg/ml	
A. ruhlandii 155B	Allicin	0.50	1
	Dimethyl thiosulfinate	2.00*	–
	Dipropyl thiosulfinate	2.00*	–
B. cenocepacia 122	Allicin	0.03	≥ 0.03**
	Dimethyl thiosulfinate	0.03	≥ 0.03**
	Dipropyl thiosulfinate	0.25	0.5
P. aeruginosa 48B	Allicin	0.06	1
	Dimethyl thiosulfinate	0.06	–
	Dipropyl thiosulfinate	0.50	–

Note. “–” – no bactericidal effect

^*^The data were obtained in the experiment on determining antibacterial activity on a solid growth medium using the disc diffusion method.

^**^But not exceeding 0.06.

The MIC and MBC values for the effect of the thiosulfinates on *B. cenocepacia *were either equal or similar, thus indicating that these two compounds exhibit a bactericidal effect. The MIC value for allicin lies in the range obtained for the activity of commercial allicin against several strains of the *B. cepacia *complex (0.008–0.062 mg/ml) [11].

**Table 2:** Antibacterial efficacy of thiosulfinates at different cell concentrations

Bacterial strain	Thiosulfinate	Diameter of inhibition zones (mm) at cell concentration, CFU/ml							
		10^4^	10^5^	10^6^	10^7^	10^4^	10^5^	10^6^	10^7^
		and thiosulfinate concentration*, mg/ml							
		2				0.4			
A. ruhlandii 155B	Allicin	30	30	30	30	0	0	0	0
	Dimethyl thiosulfinate	30	30	30	30	0	0	0	0
	Dipropyl thiosulfinate	30	30	30	30	0	0	0	0
B. cenocepacia 122	Allicin	25	25	25	25	0	0	0	0
	Dimethyl thiosulfinate	25	25	25	25	0	0	0	0
	Dipropyl thiosulfinate	20	20	20	20	0	0	0	0
P. aeruginosa 48B	Allicin	10	0	0	0	0	0	0	0
	Dimethyl thiosulfinate	15	15	15	15	10	-	-	-
	Dipropyl thiosulfinate	15	15	0	0	0	0	0	0

^*^Thiosulfinate concentrations of 0.2, 0.1, and 0.05 mg/ml are not presented in the table, since no antibacterial effect was noted at these concentrations.

Thiosulfinates exhibit a bacteriostatic effect on *P. aeruginosa *48B, since the MBC value in the studied range of thiosulfinate concentrations was determined only for allicin (1 mg/ml). The MIC and MBC values for the activity of allicin against *P. aeruginosa *48B correspond to the MIC (0.064–0.512 mg/ml) and MBC (0.128–1.024 mg/ml) values for the activity of allicin against the three clinical strains of *P. aeruginosa *[12].

**Table 3:** Resistance (+) of bacterial strains to antibiotics

Strain	Aztreonam	Amikacin	Gentamicin	Doxycycline	Imipenem	Colistin	Levofloxacin	Norfloxacin	Ofloxacin	Tobramycin	Chloramphenicol	Cefepime	Cefotaxime	Ceftazidime	Ceftriaxone	Cefuroxime	Ciprofloxacin
A. ruhlandii 155B	+		+	+	+	+	+	+	+	+	+	+	+	+	+	+	+
B. cenocepacia 122		+	+							+		+		+			+
P. aeruginosa 48B			+				+		+	+	+		+	+	+		+

The antibacterial effect of thiosulfinates on *A. ruhlandii *155B was the least significant. The MIC values obtained in the experiment for determining antibacterial activity on a solid growth medium using the disc diffusion method (*Table 2*) were 2 mg/ml for dimethyl and dipropyl thiosulfinates, which exceeded the maximum concentration used in the serial dilution experiments. Allicin was the most effective thiosulfinate against *A. ruhlandii *155B: it showed bactericidal action at a concentration of 1 mg/ml.

Changes in the antibacterial efficacy of thiosulfinates were determined depending on the concentration of bacterial cells. The experiment was carried out using the disc diffusion method (*Table 2*) and by applying samples on a solid nutrient medium. The results obtained through both methods coincided.

Thiosulfinates at a concentration of 2 mg/ml effectively inhibited the growth of *A. ruhlandii *155B and *B. cenocepacia *122 at a cell concentration ≤ 107 CFU/ml. The antibacterial effect of thiosulfinates against *P. aeruginosa *48B was quite low. Allicin at maximum concentration only slightly suppressed the growth of *P. aeruginosa *48B even at minimal cell concentration. Interestingly, it was only dimethyl thiosulfinate that, among all thiosulfinates, suppressed the growth of *P. aeruginosa *at a concentration of 0.4 mg/ml (*Table 2*). The results obtained for allicin and dimethyl thiosul finate are consistent with the data for *P. aeruginosa *from murine intestinal [7].

The absence of inhibition zones in the experiment on the solid growth medium with allicin and dimethyl thiosulfinate at low concentrations is probably due to the slow diffusion of substances into Muller-Hinton agar. Thus, the serial dilution method is the optimal technique for determining the antibacterial activity of the studied thiosulfinates.

The resistance of the *A. ruhlandii *155B, *B. cenocepacia *122, and *P. aeruginosa *48B strains was evaluated using the 17 antibiotics most commonly prescribed to patients with cystic fibrosis (*Table 3*). The *A. ruhlandii *155B strain showed resistance to 16 antibiotics, while the *B. cenocepacia *122 and *P. aeruginosa *48B strains were resistant to six and nine antibiotics, respectively. The obtained data confirmed that these strains develop resistance after prolonged antibiotic therapy. It is noteworthy that none of the antibiotics tested in this study had an antibacterial effect against all three bacterial strains.

**Table 4:** Antibacterial efficacy of thiosulfinates and antibiotics at a cell concentration of 10^6^ CFU/ml

Concentration, µg/disc	Thiosulfinate	Inhibition zone diameter, mm		
		25	20	0
20	Allicin	25	20	0
20	Dimethyl thiosulfinate	16	30	30
20	Dipropyl thiosulfinate	30	5	0
5	Imipenem	0	30	30
10	Tobramycin	0	0	0
10	Ciprofloxacin	0	0	0

We compared the efficacy of the antibacterial action of the thiosulfinates and broad-spectrum antibiotics belonging to the three different groups most commonly prescribed to patients with cystic fibrosis: imipenem belonging to the group of carbapenems, tobramycin belonging to the group of aminoglycosides, and ciprofloxacin belonging to the group of fluoroquinolones (*Table 4*). Identically to the case of two-fold serial dilutions, determination of antibacterial activity by the method of disc diffusion on a dense growth medium demonstrated that three strains were resistant to tobramycin and ciprofloxacin at standard concentrations of 10 μg/disc. The diameters of inhibition zones for *B. cenocepacia *122 and *P. aeruginosa *48B were similar to those for dimethyl thiosulfinate and slightly higher than the inhibition zone of allicin for *B. cenocepacia *122. Allicin and dimethyl thiosulfinate inhibit the growth of *A. ruhlandii *155B, while this strain is resistant to imipenem.

The obtained data open up possibilities for the development of agents for the antibacterial therapy of chronic pulmonary infections in patients with cystic fibrosis.

## Abbreviations

MGL: methionine γ-lyase
MIC: minimum inhibitory concentration
MBC: minimum bactericidal concentration
